# Nutrient Pollution: A Persistent Threat to Waterways

**DOI:** 10.1289/ehp.122-A304

**Published:** 2014-11-01

**Authors:** John Manuel

**Affiliations:** John Manuel of Durham, NC, is a regular contributor to *EHP* and the author of *The Natural Traveler Along North Carolina’s Coast* and *The Canoeist*.

Passage of the Clean Water Act of 1972 brought many improvements to surface waters by curbing much of the toxic and organic pollution going into waterways. But 42 years later, we have yet to make significant reductions in two major pollutants in our rivers, lakes, and coastal sounds—the nutrients nitrogen and phosphorus. Although nitrogen pollution overall has gone down in U.S. streams and rivers since 2004, it remains a serious problem in many waterways, and phosphorus pollution has gone up significantly.^1^ The problem is especially challenging in that the deleterious effects of nitrogen and phosphorus often occur hundreds or thousands of miles from where the nutrients originate.

**Figure:**
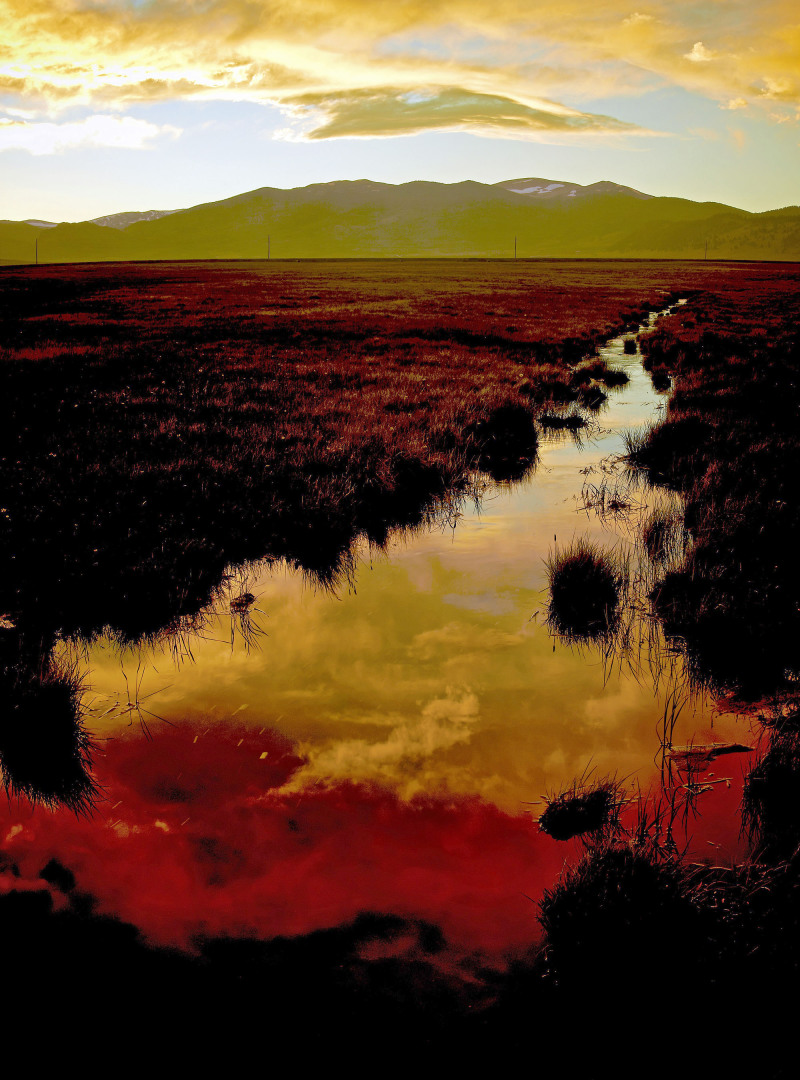
Point-source releases of nitrogen and phosphorus have declined dramatically since the 1970s, but nonpoint-source pollution continues to pose a significant threat to water quality. © Sean Brubaker/Water Rights/Corbis

Why have these two nutrients proven so tough to get under control? And are current regulatory and programmatic efforts enough to turn this situation around?

## Too Much of a Good Thing

The basics of nutrient pollution are simple enough. Nitrogen and phosphorus occur naturally in soil and water and, with respect to nitrogen, in the air we breathe. They also are added to the environment by humans, principally as fertilizers. These fertilizers enhance the growth not just of crops on land but also of algae and aquatic plants in the waters where they end up.^^2^^

Above certain levels, nitrogen and phosphorus cause algae to grow faster than ecosystems can handle. When algae die, the decomposition process consumes oxygen. Nutrient pollution also affects submerged aquatic vegetation, but in a different way: The nutrient-enriched sediment that comes off fields and impervious surfaces decreases the light available for these plants, and the shading leads to their death. Then they, too, consume oxygen as they decompose.^^3^^

Large algal blooms can entirely eliminate the oxygen in a body of water, a condition known as hypoxia that kills virtually all aquatic organisms unable to escape these so-called dead zones. According to an ongoing anaylsis by the Virginia Institute of Marine Sciences, the area of oceanic dead zones increased by one-third between 1995 and 2007.^^4^^ The hypoxic zone that forms in the Gulf of Mexico each summer varies in size from year to year but averages approximately 5,500 square miles, or roughly the size of the state of Connecticut.^^5^^

Toxins produced by harmful algal blooms (HABs) can also directly threaten human health. If ingested or contacted, these toxins can cause skin irritation, stomach cramps, vomiting, nausea, diarrhea, fever, headache, muscle and joint pain, blisters of the mouth, and liver damage.^^6^^ Local water treatment plants may not have the equipment necessary to rid drinking water of these toxins. In that case, the only safe course of action is to find other sources of drinking water for however long the toxin persists in the water supply, as was demonstrated in August 2014, when hundreds of thousands of Toledo residents found themselves without potable water.^7^ HABs can also have severe economic impacts on recreational and commercial fishing, business, and tourism. The U.S. Environmental Protection Agency (EPA) estimates that U.S. tourism alone loses close to $1 billion a year through losses in fishing and boating activities.^8^

**Figure:**
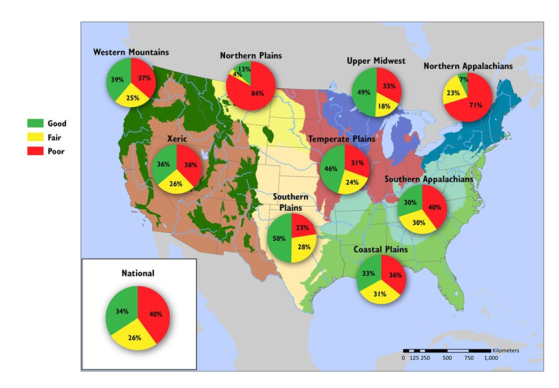
**Phosphorus Pollution in U.S. Rivers and Streams** In eight of the nine ecoregions defined by the EPA, phosphorus levels are consistently rated poor (i.e., high) in at least a third of river and stream miles. The Northern Plains and the Northern Appalachians have the highest proportions of miles rated poor (84% and 71%, respectively). The Southern Plains ecoregion has the highest percentage of river and stream miles rated good (50%), with only 23% rated poor for phosphorus levels. Source: EPA.^1^ Percents may not add up to 100% due to rounding.

According to the EPA’s latest National Rivers and Streams Assessment, some 40% of the nation’s river and stream length has elevated levels of phosphorus, and 28% has elevated levels of nitrogen, putting these waters at risk for poor quality as measured by their ability to support aquatic life.^1^ Where are the nutrients coming from? The principle source of phosphate and nitrogen is nonpoint-source pollution—the diffuse pollution from myriad inputs that accumulates into a problem at the watershed level.^9^

Although relative amounts vary from watershed to watershed, the fertilizer and animal waste that leach off farmed land generally contribute the most nonpoint-source nutrient pollution to U.S. waterways.^10^ Other nonpoint sources include stormwater runoff carrying lawn fertilizers and pet waste,^11^ and atmospheric deposition, much of it from vehicle exhaust and coal- and oil-burning power plant emissions.^12^

Individual farms also may be considered point sources of pollution, depending on what they directly discharge into waterways.^13^ Point sources of pollution are regulated by the federal government through the National Pollutant Discharge Elimination System and overall have dramatically reduced their releases of nutrients since the 1970s.^14^ However, wastewater contains large amounts of nitrogen and phosphorus from human waste, food, and some soaps and detergents, and not all of it is removed in the treatment process. Wastewater treatment plants with less advanced technology can therefore still be significant point-source contributors of nutrient pollution.^15^

## The TMDL Approach

With such widespread pollution caused by so many different sources, it’s no wonder the United States is challenged politically, technologically, and financially to solve the problem of nutrient pollution. The Clean Water Act of 1972 and its various amendments set numeric limits for a variety of chemical pollutants emitted from point sources. However, phosphorus and nitrogen are not among the regulated chemicals. Furthermore, the law does not include regulation of nonpoint-source pollution.

Section 303(d) of the Clean Water Act does require states to submit a list of impaired and threatened waters within their jurisdiction and establish priorities for the development of total maximum daily loads (TMDLs) of pollutants for these water bodies. A TMDL is a calculation of the maximum amount of a pollutant that a water body can receive and still meet federal water quality standards. The TMDL is tailored to reflect how that specific water body is used. For example, a lake used for drinking water might have more stringent limits on phosphorus than one used just for recreation. Thus, while there are no overall federal limits on nitrogen or phosphorus pollution, these nutrients can be managed as part of a TMDL implementation plan.^16^

The TMDL approach was largely overlooked in the 1970s and 1980s as governments focused on bringing point sources into compliance with the Clean Water Act. More recently, however, attention has turned to the establishment of TMDLs to address other sources of pollution.

But the steps involved in developing a TMDL are time-consuming and costly. States must first identify waters not in compliance with the Clean Water Act, then prioritize water bodies for the development of TMDLs. Due to a lack of money and personnel, most state agencies are able to monitor only a small percentage of their waters consistently enough to detect water-quality problems.^15^

A third step involves developing a TMDL for each pollutant. This step can take years, especially for a large water body like the Chesapeake Bay, whose watershed encompasses 64,000 square miles in six states and the District of Columbia. Stakeholders in affected jurisdictions meet to hammer out goals, actions, and timetables. Proposed limits must be submitted to the EPA for approval. Planning for the Chesapeake Bay TMDL began in 2000 and was not approved by the EPA until December 2010.^17^ (The Bay TMDL is actually a combination of 92 smaller TMDLs for individual Chesapeake Bay tidal segments.^16^)

**Figure:**
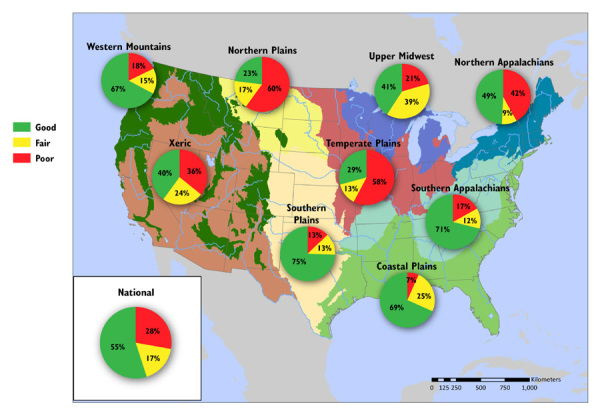
**Nitrogen Pollution in U.S. Rivers and Streams** Many regions show less severe impacts from nitrogen than from phosphorus. The highest proportions of miles in poor condition for nitrogen are found in the Northern Plains (60%), the Temperate Plains (58%), the Northern Appalachians (42%), and the Xeric (36%). In four ecoregions (Coastal Plains, Southern Plains, Southern Appalachians, and Western Mountains) the majority of river and stream miles are rated good for nitrogen. Source: EPA.^1^ Percents may not add up to 100% due to rounding.

Finally, the TMDL must be implemented. Again, this can take years following the EPA’s approval of a plan, with pollution reduction goals being targeted in stages. With respect to the Chesapeake Bay’s TMDL, 60% of the plan’s goal for reducing nutrients and sediment is anticipated to be met by 2017, and 100% should be met by 2025.^18^ As with most complex plans of this nature, however, actual implementation may take much longer, and the costs can be staggering. For instance, estimates for the state of Maryland to fully implement its portion of the Chesapeake Bay TMDL total $928 million for farmers, $2.37 billion for municipal wastewater systems, $7.39 billion for stormwater systems, and $3.72 billion for septic tank upgrades.^19^

## Best Management Practices

TMDLs are not the only vehicle being used to address nutrient pollution. Federal programs including the Conservation Reserve Program (CRP), administered by the U.S. Department of Agriculture, provide direct rental payments to farmers who remove environmentally sensitive acreage from agricultural production and implement conservation practices.^20^ The EPA awards grants to states to build or upgrade wastewater treatment plants and to support various state-level nonpoint-source management programs.

Various best management practices (BMPs) are being employed to reduce nutrient pollution from urban sources. Technologies such as detention basins, constructed wetlands, vegetative swales, and bioretention facilities (e.g., rain gardens) can all be used to slow down stormwater and biologically degrade the nutrients before they reach waterways. Practices that reduce nutrient runoff from developed areas include leaf collection in the fall, bagging of dog waste, and prohibitions on phosphorus in lawn fertilizers.^21^ Agriculture employs a whole different array of proven BMPs ranging from planting cover crops in winter, to better timing and amounts of fertilizer application, to the establishment of vegetated buffers along streams.^22^

Yet, even after decades of research, much remains unknown about how phosphorus and nitrogen interact in the environment. For instance, recent studies in Lake Superior suggested that reducing phosphorus loads may actually lessen the ability of aquatic organisms to remove nitrogen from the water. The authors pointed out this should “in no way be considered as a rationale for relaxing [phosphorus] control measures.”^23^ Instead, they wrote, the results suggest more attention should be paid to controlling nitrogen in tandem with phosphorus—which will be challenging, they added, given that sources of nitrogen tend to be even more diffuse than those of phosphorus.^22^

With respect to farmers, the emphasis has been on use of incentives to encourage voluntary adoption of less-polluting practices. These approaches commonly use financial, educational, and technical assistance as a stimulus. However, surveys suggest that in key farming states such as Iowa, overall participation is low, and among farmers who do participate, the investment in conservation tends to be small.^24^ Referring to a 2011 poll of Iowa farmers, a report by the nonprofit Iowa Policy Project noted that 51% of respondents reported making no conservation expenditures in the past 10 years, and more than one-third were unaware of many of the conservation programs available in the state.^25^

The report authors further pointed out that farmers were enrolling fewer of their acres in the federal CRP. “Iowa CRP acres are decreasing, falling by almost one-fourth, from 1,970,486 acres in 2007 to 1,525,012 in 2012,” they wrote. “The drop in CRP enrollment has coincided with the ethanol boom and the rise in the price of corn, suggesting that the economic bottom line does affect a farmer’s willingness to adopt conservation measures. When [government] subsidies pay less than cash rent, the conservation practices disappear.”^24^

**Figure:**
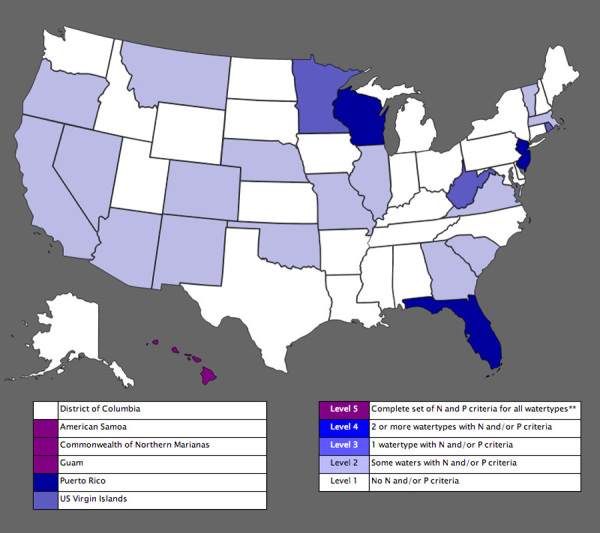
**States with Numeric Criteria for Total Nitrogen (N) or Total Phosphorus (P)** This map shows a national summary of current numeric total nitrogen and total phosphorus EPA-approved criteria. More criteria are expected to be added in the near future, according to state-provided information. ”Watertypes” refers to three types of water bodies: lakes/reservoirs, rivers/streams, and estuaries. Source: EPA^28^

Nutrient trading between point and nonpoint sources is an idea that is promoted as an alternative to cost-sharing. In this voluntary system, farmers accumulate and sell credits by implementing conservation measures that reduce nutrient loads. Wastewater treatment plants in the same watershed buy the credits from farmers instead of investing in new technology to meet federal requirements for reducing nutrient output.^26^

Nutrient trading has worked well in the Long Island Sound, where there are many wastewater treatment plants and farms in the same watershed.^27^ Patrick Parenteau, a professor of law and senior counsel to the Environmental and Natural Resources Law Clinic at Vermont Law School, notes this program currently operates between point sources, mainly publicly owned treatment works in Connecticut. “There has been talk about including nonpoint sources,” he says, “but it hasn’t gotten there yet.”

But in other watersheds such as the Maumee Valley in western Lake Erie, farms contribute vastly more nutrients than do the few wastewater treatment plants, so opportunities for trading are limited. Further, nutrient trading programs can be complex, and they take time to establish.

“There is a lot of both hope and frustration [among farmers] with nutrient trading,” says John Bell, government affairs counsel for the Pennsylvania Farm Bureau. “Pennsylvania set a reasonable set of ground rules for nutrient trading, but even with this, it’s hard for farmers to get enthusiastic because of the limited credit given to their conservation practices.” He explains that a farmer may implement a practice that reduces a hundred pounds of nitrogen at the stream flowing past his farm, but will only receive nutrient trading credit for the impact that action has in waters possibly hundreds of miles away. “Very few practices to reduce nonpoint-source pollution have an immediate impact on a watershed,” he says. “Often, the impacts are not measurable for a number of years after the [practice] was first implemented.”

Some experts believe that without setting numeric water-quality standards for nitrogen and phosphorus, efforts to combat nutrient pollution will fail. For now, almost half the states have established statewide numeric limits on nitrogen and/or phosphorus in at least some water bodies. Hawaii is the only state with a complete set of nitrogen and phosphorus criteria for all types of water bodies.^28^ Whether these states are able to maintain and enforce meaningful standards remains to be seen.
