# Erratum: “Nutrient Pollution: A Persistent Threat to Waterways”

**DOI:** 10.1289/ehp.122-A323

**Published:** 2014-12-01

The November 2014 News article “Nutrient Pollution: A Persistent Threat to Waterways” [Environ Health Perspect 122:A304–A309; http://dx.doi.org/10.1289/ehp.122-A304] accidentally used the same map to depict both phosphorus and nitrogen pollution in U.S. waterways. In addition, the caption for the phosphorus map incorrectly stated that six of nine ecoregions have phosphorus levels rated “poor” in at least a third of river and stream miles, when in fact this is true of eight ecoregions.

*EHP* regrets the errors.

